# High-throughput and single-cell imaging of NF-κB oscillations using monoclonal cell lines

**DOI:** 10.1186/1471-2121-11-21

**Published:** 2010-03-16

**Authors:** Sina Bartfeld, Simone Hess, Bianca Bauer, Nikolaus Machuy, Lesley A Ogilvie, Johannes Schuchhardt, Thomas F Meyer

**Affiliations:** 1 Max Planck Institute for Infection Biology, Department of Molecular Biology, Berlin, Germany; 2 MicroDiscovery GmbH, Berlin, Germany; 3 Hannover Medical School (MHH), 30625 Hannover, Germany

## Abstract

**Background:**

The nuclear factor-κB (NF-κB) family of transcription factors plays a role in a wide range of cellular processes including the immune response and cellular growth. In addition, deregulation of the NF-κB system has been associated with a number of disease states, including cancer. Therefore, insight into the regulation of NF-κB activation has crucial medical relevance, holding promise for novel drug target discovery. Transcription of NF-κB-induced genes is regulated by differential dynamics of single NF-κB subunits, but only a few methods are currently being applied to study dynamics. In particular, while oscillations of NF-κB activation have been observed in response to the cytokine tumor necrosis factor α (TNFα), little is known about the occurrence of oscillations in response to bacterial infections.

**Results:**

To quantitatively assess NF-κB dynamics we generated human and murine monoclonal cell lines that stably express the NF-κB subunit p65 fused to GFP. Furthermore, a high-throughput assay based on automated microscopy coupled to image analysis to quantify p65-nuclear translocation was established. Using this assay, we demonstrate a stimulus- and cell line-specific temporal control of p65 translocation, revealing, for the first time, oscillations of p65 translocation in response to bacterial infection. Oscillations were detected at the single-cell level using real-time microscopy as well as at the population level using high-throughput image analysis. In addition, mathematical modeling of NF-κB dynamics during bacterial infections predicted masking of oscillations on the population level in asynchronous activations, which was experimentally confirmed.

**Conclusions:**

Taken together, this simple and cost effective assay constitutes an integrated approach to infer the dynamics of NF-κB kinetics in single cells and cell populations. Using a single system, novel factors modulating NF-κB can be identified and analyzed, providing new possibilities for a wide range of applications from therapeutic discovery and understanding of disease to host-pathogen interactions.

## Background

NF-κB is a family of transcription factors that plays a critical role in regulating genes involved in cell proliferation, apoptosis, innate immunity, and inflammatory responses. NF-κB is activated by a range of physical and chemical signals including cytokines and bacterial (e.g. lipopolysaccharide; LPS) and viral products [1,2]. Deregulation of the NF-κB system is implicated in many diseases including cancer [[3,4], http://www.nf-kb.org]. Understanding the specificity and the temporal nature of NF-κB responsive gene expression is therefore not only of physiological interest but of crucial clinical importance.

Functional NF-κB is assembled through homo- or heterodimerization of the five subunits: p65 (RelA), RelB, c-Rel, p105/p50 (*nfkb1*) and p100/p52 (*nfkb2*). The classical dimer p65:p50 is ubiquitously expressed and is the primary mediator of inflammation [2]. NF-κB dimers reside in the cytoplasm sequestered by inhibitor proteins, e.g. IκBα (Inhibitor of κBα), until a signal cascade is activated that leads to phosphorylation, ubiquitinylation and subsequent degradation of the inhibitor. As a result, the dimer translocates into the nucleus where it binds to DNA and triggers transcription of target genes. One of the target genes is the inhibitor itself creating a negative feedback loop. If the stimulus remains, repeated inhibitor degradation and re-synthesis can lead to oscillations of nuclear translocations. Further regulation mechanisms dampen the oscillations and finally lead to termination of the signaling cascade [2,5-7].

Oscillations of NF-κB subunit nuclear translocation have been observed in single cells using fluorescence live-cell imaging [8-11]. Additionally, similar repeated cycles of DNA binding activity have been shown using the electrophoretic mobility shift assay (EMSA) in a range of cell types [12,13]. In cell populations oscillations can be asynchronous due to phase interval heterogeneity in single cells [8,9]. Dynamics of nuclear translocation or DNA binding can vary in response to different stimuli: While a sustained TNFα stimulus induces oscillations, a short pulse of TNFα leads to a single peak of activation [14]; LPS elicits, via secretion of TNFα and the subsequent overlap of the two signaling pathways (LPS and TNFα), a very heterogeneous response, with some cells displaying only one cycle of nuclear translocation, while other cells display oscillations or persistent nuclear translocation [11,15,16]. These observations have raised the possibility that NF-κB activation dynamics could determine gene activation specificity. Indeed, different frequencies of stimulation with TNFα induce different gene expression patterns [9,12].

While oscillations have been shown in response to TNFα [8-15,17,18], the topoisomerase II inhibitor etoposide [8] and LPS [11] they have not yet been investigated during infection with live bacteria. The observation that oscillations occur in response to bacteria - being single units that naturally occur in infections - would be an important indication that they are physiologically relevant processes.

Although there are several methods to measure NF-κB activation, only some are suitable for investigating the dynamics of this signaling system. Commonly used tools for general NF-κB analysis are reporter plasmids, in which an NF-κB binding site drives the transcription of reporter genes like enhanced green fluorescent protein (EGFP) or luciferase. Stable cell lines with these constructs are commercially available (i.e. from Invitrogen) and similar constructs have been successfully used in analysis of other oscillators such as the circadian clock [19]. However, these constructs and cell lines measure transcriptional responses accumulated after activation and therefore do not necessarily reflect the immediate translocation of NF-κB subunits to the nucleus in real time. In addition, many reporter constructs respond to several NF-κB dimers and do not report the activity of one particular NF-κB dimer.

Biochemical approaches like EMSA have been used successfully to detect oscillations in the temporal response of NF-κB [12,15,16,18]. These experimental observations have also been simulated in a multivariable mathematical model of NF-κB activation dynamics [12]. Although useful, biochemical approaches are time consuming and the protein extracts used for analysis average the potentially asynchronous responses of single cells.

Since activation of NF-κB is marked by the translocation of NF-κB subunits from the cytoplasm to the nucleus, many groups have used high-content image analysis of fixed and immunostained cells [20-22]. Other studies have followed the dynamics of NF-κB activation in live cells using fluorescent protein fusions [8-11,14,23-26]. Using the latter technique, Nelson and co-workers were the first to show NF-κB oscillations at a single-cell level [8,9].

Here, we combined high-content automated fluorescence imaging of NF-κB protein subunit fusions with the stability of monoclonal cell lines to analyze the real-time dynamics of NF-κB activation within single cells. We have generated lentivirally transduced monoclonal cell lines expressing p65 fused to GFP that can be used in any microscopic analysis including high-throughput automated microscopy and live-cell imaging. Using these cell lines, we show inducer-specific temporal control of p65-translocations and, for the first time, oscillations in response to infection with the bacterium *H. pylori*. These experimental data were also used to construct a simplified mathematical model of NF-κB signaling regulation.

## Results

### A high-throughput assay for monoclonal cell lines expressing p65-GFP

**Figure 1:**
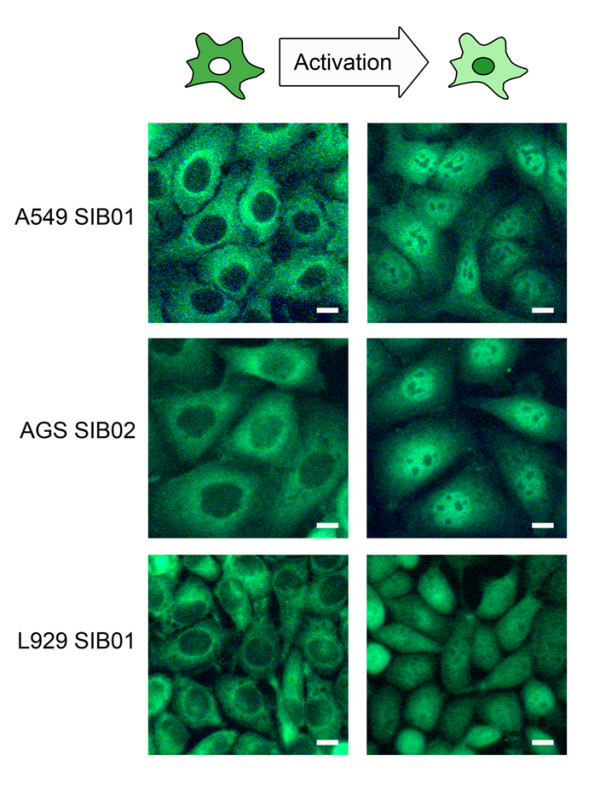
**p65-GFP reporter cells A549 SIB01, AGS SIB02 and L929 SIB01**. Cells were seeded in 96-well plates and either left untreated (non-activated) or activated with TNFα (10 ng/ml) for 30 min. Cells were fixed and pictures were taken with a Leica DMR Microscope. Representative cells are shown. Scale bar = 10 μm.

To analyze p65 nuclear translocation within a large number of cells, we developed an automated high-throughput assay using image analysis. First, we generated monoclonal p65-GFP expressing variants of the human alveolar epithelial cell line A549 (A549 SIB01), the human gastric epithelial cell line AGS (AGS SIB02) and the mouse fibroblast cell line L929 (L929 SIB01) via lentiviral transduction. Expression of p65-GFP in comparison to that of endogenous p65 was similar in A549 SIB01, but increased sevenfold in the AGS SIB02 and L929 SIB01 cell lines (Additional file 1). To determine the number of virus integration sites, the long-terminal repeat (LTR) region (which borders the integration site) of the integrated lentivirus was PCR amplified. The A549 SIB01, AGS SIB02 and L929 SIB01 cell lines contained approximately four, six and ten integration sites, respectively. Successful sequencing of six sites revealed most were located in non-coding sequences and thus had no obvious influence on NF-κB signaling (Additional file 2). Using various inducers, we then tested the utility of the cell lines to detect nuclear translocation of p65. In non-stimulated cells, p65-GFP was mainly localized to the cytoplasm, whereas upon stimulation with TNFα (10 ng/ml), p65-GFP translocated into the nucleus (Figure 1).

**Figure 2:**
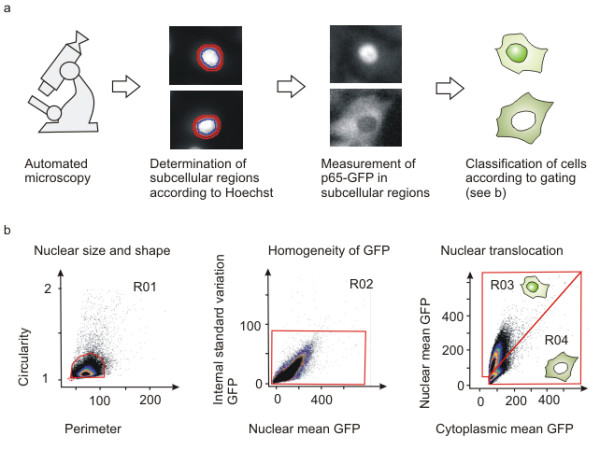
**The NF-κB readout: p65-GFP reporter cells A549 SIB01 and automated microscopy. a) **Workflow of automated microscopy and picture analysis. Cells are fixed and stained with Hoechst 33342. Pictures are then acquired with the Scan^R system and nuclear (blue) and cytoplasmic (red) areas are defined in the Scan^R software. One activated (top) and one non-activated cell (bottom) is depicted. **b) **Translocation assay using Scan^R Analysis. Cells were seeded on 96-well plates, activated with TNFα (10 ng/ml), fixed, stained with Hoechst 33342 and then analyzed with automated microscopy. Scatter plots as depicted in the analysis software are shown. Cells are gated for circularity and size (Region R01), intensity of GFP and standard deviation of GFP intensity (Region R02). Cells in regions R01 and R02 are classified as active or inactive according to nuclear and cytoplasmic GFP intensity (Region R03 or R04). Cells with nuclear p65-GFP are also in region R03, whereas cells with mainly cytoplasmic p65-GFP are also in gate R04.

For the automated image analysis assay, cells were seeded into 96-well plates, stimulated for the optimized time, fixed and nuclei stained with Hoechst. Images of cells were acquired using automated microscopy. Cell nuclei were detected and the surrounding cytoplasmic area set using image analysis software (Figure 2a). To provide quantitative analysis of the nuclear translocation of p65, cells were then depicted on dot plots and gated according to various predefined parameters: We used perimeter and circularity of nuclei to gate for nuclear size and circularity, standard deviation of GFP signal to gate for homogeneity of GFP signal, and intensity of nuclear and cytoplasmic GFP to gate for cells with nuclear p65 (Figure 2b). Numbers of cells with mainly nuclear p65 or cells with mainly cytoplasmic p65 (usually 100-1000 cells per well) were counted and percentages of cells with mainly nuclear p65 per well calculated (Figure 2 and Additional file 3). This threshold-based assay gave the clearest and simplest representation of the nuclear translocation. Furthermore, applying this read-out, the distribution of the response can also be displayed, giving a more detailed representation of intermediate translocations (Additional file 4).

### Comparison of parental and p65-GFP cell lines

Mathematical models have predicted that ectopic expression of NF-κB subunits could alter the dynamics of the signaling module [27], while experimental data have shown that this is not necessarily the case [28]. Therefore, we compared NF-κB activation in parental, non-transduced cell lines with p65-GFP expressing cell lines. Western blot analysis showed both cell lines exhibited very similar patterns of IκBα degradation over a period of 90 minutes after stimulation with TNFα at 10 ng/ml or 0.5 ng/ml (Additional file 5a). However, while the overall degradation pattern is comparable, the p65-GFP expressing cell lines seem to be slightly faster in restoring IκBα to baseline levels (i.e. non-stimulated sample levels). Therefore, we do not completely exclude the possibility that expression of the p65-GFP fusion slightly alters signaling dynamics. The degradation of IκBα in parental and p65-GFP expressing cell lines corresponded well to the percentages of cells with nuclear p65 calculated using the automated p65-translocation assay (Additional file 5b).

### Stimulus-specific temporal control of p65-GFP translocations

**Figure 3:**
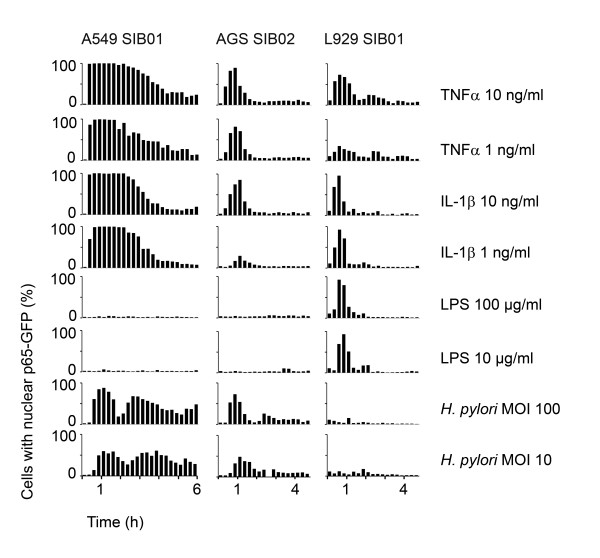
**Inducer-specific activation profiles of cell lines A549 SIB01, AGS SIB02 and L929 SIB01**. The cell lines were activated with the indicated inducer for times between 0 and 6 h: A549 SIB01 was activated for 0-345 min, AGS SIB02 and L929 SIB01 for 0-270 min, each at intervals of 15 min. Cells were fixed, stained with Hoechst 33342 and then analyzed with automated microscopy and image analysis software. For each cell line, single cells were analyzed and the mean percentage of cells with nuclear p65-GFP per well was calculated as described in Figure 2. Mean percentages from duplicate experiments are shown as bars. Results are representative of at least three independent experiments. Standard deviations are not shown for graphical reasons. One data point is missing in AGS *H. pylori *MOI 10 due to technical issues.

Using the monoclonal p65-GFP cell lines, it became apparent very quickly that there was no uniform response to TNFα, but that each cell line had a distinctive pattern of NF-κB activation. To further characterize this specificity, we analyzed the response of the cell lines to different inducers: the cytokines TNFα and interleukin 1β (IL-1β), the bacterial cell wall component LPS and the bacterium *H. pylori *(Figure 3). Cells were seeded in 96-well plates, activated by the respective inducer, fixed after the indicated time and p65-translocation was quantified by automated microscopic and image analysis.

The resulting profiles were highly specific regarding cell lines and inducers (see Figure 3). The main characteristics were: (i) The ability to recognize a stimulus depended on the cell line. None of the human epithelial cell lines tested responded to LPS, whereas the mouse fibroblasts were unresponsive to *H. pylori*, suggesting strict stimulus specificity, probably due to differences in functional receptors or signaling pathways present in the respective cells. (ii) Temporal profiles were stimulus specific. A549 exhibited damped oscillations after infection with *H. pylori*, but stable translocation after stimulation with TNFα or IL-1β. Stable translocations in response to TNFα were verified by live-cell imaging of A549 cells (data not shown). (iii) The percentage of activated cells, but not the temporal profile, was variable to the dose of the inducer. A reduction in the dose of IL-1β in AGS or TNFα in L929 led to a reduction in percentage of activated cells. (iv) Thresholds were cell-line specific. Low-dose challenge of AGS with IL-1β produced low percentages of activated cells, while the same doses yielded a full response in A549 and L929 cell lines. (v) The duration of the response varied between cell lines. A549 generally showed much longer responses, implying more amplification of the signal or fewer dampening mechanisms in this cell line. (vi) Only minimal background p65-translocation activity was observed in all cell lines (i.e. no stimulus, time 0).

### Bacterial infection induces oscillations of p65-GFP

We observed damped oscillations of p65 translocation in response to *H. pylori *using the high-throughput assay (Figure 3). This bacterium is equipped with a type four secretion system (TFSS) capable of injecting its cytotoxicity associated protein A (CagA) as well as bacterial peptidoglycan into its infected host cell. Consistent with previous studies that employed a variety of techniques to analyze *H. pylori-*induced NF-κB activity [29-32], the oscillations shown here were dependent on a functional TFSS but not on CagA or other factors possibly secreted into the medium by bacteria or cells (Additional file 6).

**Figure 4:**
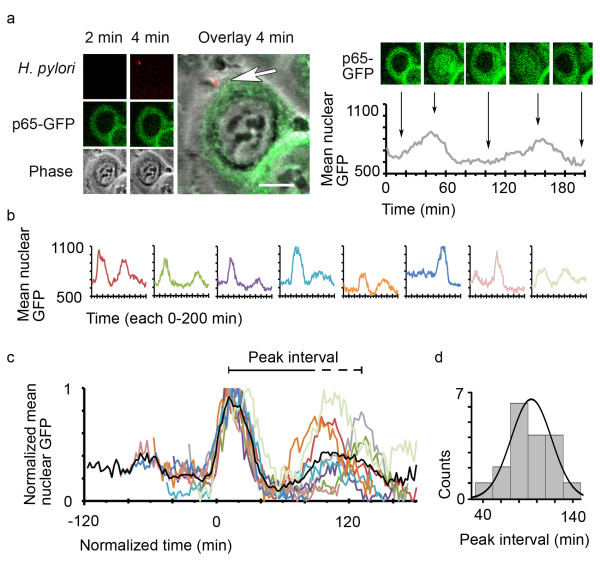
***H. pylori *induces damped oscillations of p65 nuclear translocations**. **a) **p65-GFP expressing AGS SIB02 cells were infected with *H. pylori*, stained with Syto 61 and analyzed by confocal live-cell microscopy. The upper panel shows a single bacterium attaching to a cell shortly after acquisition begins (arrow indicates position of bacterium). Graph shows average intensity of GFP in a representative nuclear region of this cell. Scale bar = 10 μm. **b) **Graph shows average intensities of GFP in other cells from the same experiment, to which one or more bacteria attached at different time points. **c) **Alignment of normalized average intensities of GFP within representative nuclear regions of the nine single cells shown in (a) and (b). Mean GFP intensity of these nine cells is shown as a black line. While the first peak is remarkably similar, the peak interval and the second peak differ between cells. **d) **Peak interval of oscillating cells ranges from 40 and 140 minutes; intervals between 80-100 minutes were most frequent. Cells were treated as in (a) and 18 oscillating cells from four separate experiments were analyzed (see Additional file 8 for details).

Oscillations visible in cell populations (as in Figure 3) can result from either damped oscillations on the single-cell level or a high cell-to-cell heterogeneity, with some cells showing only one cycle of transclocation. To investigate this question, we analyzed bacterial infections on a single-cell level using live-cell imaging. AGS SIB02 cells were infected with *H. pylori *at a multiplicity of infection (MOI) of 5 and stained with a live dye. Interestingly, cell attachment of a single bacterium appeared to induce full p65-GFP translocation (Figure 4a). To characterize any induced oscillations, we compared the average GFP intensities of nine cells from one experiment. Individual cells were activated at different time points, each roughly 20 min after the attachment of one or more bacteria (Figure 4b). When we mathematically aligned the different oscillations for the first peak, remarkable features became apparent: While the first peak seems fairly synchronous in all cells, the second peak and its interval are variable (Figure 4c). Analysis of a further 33 cells under various experimental conditions (MOI and time), revealed that while the majority of cells exhibited the expected damped oscillations (high first peak followed by smaller second peak), the opposite was also possible (lower first peak followed by higher second peak) (Additional file 7). Peak intervals measured in 18 cells were between 40 and 140 minutes, with the most frequent intervals being between 80 and 100 minutes (Figure 4d). Taken together, our observations reveal a moderate cell-to-cell variability in *H. pylori-*induced p65 nuclear translocation oscillations.

### Modeling oscillations after bacterial infection

**Figure 5:**
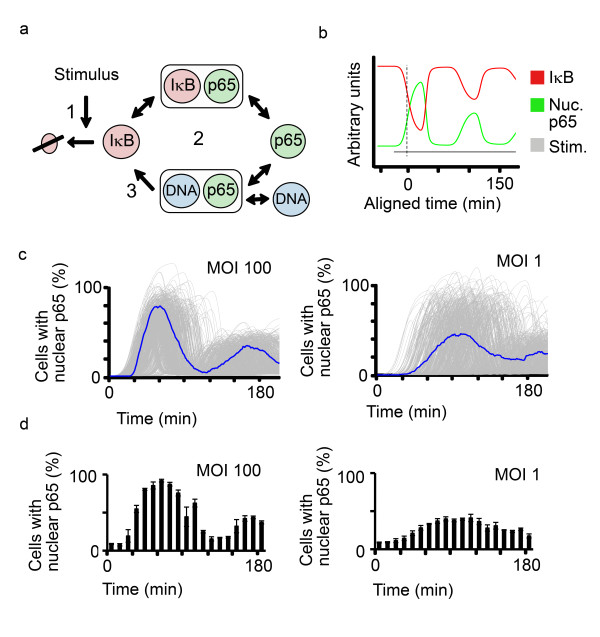
***H. pylori*-induced oscillations of p65 nuclear translocation at the population level are dependent on MOI in simulations and experiments**. **a) **Mathematical model for simulating p65 oscillations: Stimulus induces degradation of IκBα (1), IκBα competitively inhibits binding of p65 to DNA (2) and transcription and translation is simplified by an activation delay (3). **b) **Mathematical model predicts oscillations of nuclear p65 (green) and protein levels of IκBα (red) upon stimulation (grey) at the single-cell level. **c) **Simulation of population-level p65 oscillations after *H. pylori *infections at an MOI 100 and 1. Oscillations of p65 nuclear translocation within 500 individual cells were simulated (grey lines) and percentages of cells with nuclear p65 (blue line) were determined by a threshold set for nuclear p65. **d) **Experimental confirmation of predicted population-level oscillations of p65 (at MOI 100 and 1). AGS SIB02 cells were infected with *H. pylori *at the indicated MOI, fixed, analyzed by automated microscopy and then the percentage of cells with nuclear p65-GFP was determined, as described in Figure 2. Error bars = SD of experiment performed in triplicates. Results are representative of three independent experiments.

To address these differential responses (Figures 3 and 4), we then used our experimental data to develop a one-variable model of temporal regulation of NF-κB signaling (see Additional file 8). While previous approaches model transcription regulation in more detail [9-12], our model simplifies the process of protein synthesis by using a time delay to describe the effect of NF-κB-induced transcription on IκBα protein level (Figure 5a). This model was able to predict damped nuclear translocation at the single-cell level (Figure 5b). We also integrated our observations that activation of single cells do not necessarily occur simultaneously (Figure 4) and thus could influence dynamics visible at the population level. We know that p65 translocation relies on contact between bacteria and cells. These activations comprise discrete events dependent on the time required by bacteria in solution to reach cells. Accordingly, at a high MOI, initial contact events will occur in a rather synchronized manner, while at a low MOI, initial contacts will occur more asynchronously. Approximating the variability in activation onset, we simulated population dynamics for bacterial infections with an MOI 100 and 1: The former led to an almost synchronized activation in which oscillations were also visible at the population level, whereas the latter resulted in masking of oscillations at the population level (Figure 5c). Most importantly, we could confirm the simulation with experimental data using AGS SIB02 cells and automated microscopy (Figure 5d), thus highlighting the power of this cell system to unify population and single cell analyses.

## Discussion

Here we describe a simple and cost-effective method amenable to high-throughput applications that combines the use of p65-GFP expressing monoclonal cell lines with automated microscopy and analysis to provide synchronous real-time analysis of NF-κB activation dynamics at a single-cell and population level. By applying this system, we reveal a single bacterium is apparently sufficient to induce translocation of p65-GFP. Moreover, we show single cellular variabilities in NF-κB dynamics, indicating the interplay of multiple parameters in the regulation of NF-κB.

There are many different oscillatory systems [33] and expression of fluorescent fusion proteins has been a useful tool to study dynamics of these oscillatory, and also non-oscillatory, signaling systems [8-11,34-38]. Oscillations in NF-κB signaling have previously been shown using a variety of techniques and cell types [9-16]. To analyze oscillations on a single-cell level, most previous studies have relied on transient protein expression [8,9] and discrepancies in oscillation synchrony between population and single-cell analysis has been discussed to result from different expression levels of the fusion protein in single cells [27]; however, experimental data has shown this is not necessarily the case [28]. Most importantly, NF-κB oscillations and heterogeneity in peak intervals are comparable between different studies using transient expressions [8,9], clonal cell lines (in which all cells have the same expression level) [9,11], stable expression on the endogenous level [11] or a GFP-p65 knock-in mouse model with endogenous p65 levels [10]. Moreover, the reported oscillations match the moderate asynchrony observed here (see Figure 4).

Advantages of the assay systems presented here are the lentiviral transduction of the p65-GFP expressing gene and its stable integration into the cellular genome. This not only avoids continued selection pressure for maintaining gene expression but allows the establishment of well-defined cell lines. Virus integration into the host cell genome is statistically unlikely to disrupt any relevant gene functions; however, this can be ascertained by sequencing the integration sites. Consistently, a comparison with the parental cells showed little influence of ectopic expression at the level of IκBα degradation (Additional file 5). Moreover, the assay system requires no extra material, avoiding unnecessary addition of experimental variables and making it highly cost-effective. Lastly, the method exhibits high sensitivity as background activity levels are very low (Figure 3).

The p65-GFP carrying lentivirus itself also offers interesting features for future approaches: Not only can it be used to generate any other cell line desired but, as lentiviruses can even infect non-dividing cells, it can be applied to the transduction of primary cells. Notably, one can influence the expression level of p65-GFP by varying the copy number of viruses per cell, i.e. using higher virus titers would lead to high numbers of integrated copies per genome and thus to higher expression levels.

Here, we observed inducer- and cell line- specific signatures of NF-κB response patterns (see Figure 3). The p65-GFP protein exhibited either oscillating translocation or rather stable activation; the percentage of activated cells varied with the dose of the inducer. Both observations are generally consistent with previous findings [15,16,39]. Stimulus-specific temporal control of NF-κB activation has also been observed in mouse embryonic fibroblasts (MEFs) using EMSAs: when stimulated with TNFα they displayed oscillatory behaviour, but when stimulated with LPS they displayed stable behaviour. The stable activation could be attributed to a positive feedback that leads, via the secretion of TNFα, to an overlap of two signaling pathways, both oscillatory when isolated but leading to stable activation when overlapping [15,16]. Live-cell analysis of single cells revealed that the stable activation observed on the population level was due to the highly heterogenous responses of single cells resulting from a noisy paracrine TNFα signal [11]. Possibly a similar mechanism lays beneath the stable activation observed here after TNFα or IL-1β induction, but as TNFα leads to a single translocation in A549 with the stimulus remaining in the medium, it seems unlikely that a potential feedback loop in this case involves TNFα. The same applies to the stimulation with IL-1β.

NF-κB oscillations have been observed in cell culture models using relatively high doses of inducers such as TNFα [8-12,14,15,17,18] and LPS [11]. Data presented here show, for the first time, oscillations in response to bacterial infection and that a single bacterium seems to be capable of inducing p65 translocation. Therefore, oscillations are likely to occur in infections in vivo. As with other inducers of NF-κB such as TNFα [8-11,14], *H. pylori *infection elicited a moderate cell-to-cell variability in p65 oscillations (Figure 4). Future experiments will clarify whether the observed variations of peak intervals in single-cell oscillations are due to stochastic transcription of the IκBα gene, as suggested recently [9], or can be attributed to the strength of induction and/or repeated infection by individual bacteria, as likely found during bacterial infection in vivo.

Based on these experimental data, we developed a simple mathematical model of NF-κB signaling temporal regulation. While our model of the NF-κB cascade may appear somewhat simplifying and might have been formulated in a much more elaborate fashion [2,12,28], we were not aiming for a detailed representation of the mechanisms or potential mechanisms leading to oscillations. Rather our goal was, given the experimental fact of oscillations, to correlate single-cell observations and population events derived from two different experimental approaches [21]. Mathematical modeling will be an important tool for linking observations on different levels of granularity and using different technologies [40]. In our situation a more complicated model of the single cell probably would not improve our understanding of the collective observations. Integrating our observations that oscillations of single cells can be masked at a population level by asynchronous stimulations, each stimulation being a discrete event dependent on MOI, we demonstrated the unique ability of our cell model to indicate differential dynamics at the population and single-cell levels. Data generated using the clonal cell lines could be used to refine and broaden our and similar mathematical models by providing experimental details of population and single-cell level oscillations within different cell types.

## Conclusions

Owing to the crucial roles that NF-κB plays in inflammation, immunity and cancer, both the pharmaceutical industry as well as research groups are actively pursuing the discovery of new compounds that modulate NF-κB [41]. The cell lines and the high-throughput microscopic assay presented here could be of considerable value for these efforts, providing a highly cost-effective method to identify and further analyze promising novel factors using one system; for instance, an RNAi-based screen could identify factors that prolong or inhibit dampening of oscillations, thereby opening new avenues of therapeutic discovery and understanding of disease. Analysis of temporal profiles of NF-κB activation in infections can unravel sequential activations and even provide indications for the inducing stimulus [42]. Furthermore, the combination of novel live-cell imaging technologies with mathematical modeling at the single-cell and cell-population level may lead to a better mechanistic understanding of the host-pathogen interaction governing infections.

## Methods

### Lentiviral vector construction and transformation

p65-GFP was a kind gift from Johannes A. Schmid [26] and was cloned into pWPXL (kindly provided by Didier Trono http://tronolab.epfl.ch/) using the primers 5'-AATAATCGACGCGTCGATGGTGAGCAAGGGCGA-3' and 5'-ACCACCCACTAGTGTTAGGAGCTGATCTGACTCAG-3'and MluI and SpeI sites. GFP used is the eGFP from Clontech. The resulting construct pSIB02 has been confirmed by sequencing. Lentiviral vector particles were generated by transient transfection of 293T using envelope and packaging vectors pMD2.G and psPAX2 (also kindly provided by Didier Trono). Virus particles were harvested from the supernatant 48 h post transfection and concentrated by ultracentrifugation at 25 000 rpm. AGS (ATCC CRL 1739, human gastric adenocarcinoma epithelial cell line), A549 (ATCC CCL-185, human lung carcinoma epithelial cell line) and L929 (ATCC CCL-1, mouse fibroblast cell line) were infected with lentiviral particles. Three days post infection, cells were singularized by serial dilutions, separation was confirmed by eye and monoclonal cell lines AGS SIB02, A549 SIB01 and L929 SIB01 were propagated.

### Virus integration sites

DNA was isolated using DNeasy tissue kit (Qiagen) according to the manufacturer's instructions. For determination of the virus integration sites, the LTR region (which borders the integration site) of the integrated lentivirus was linearly amplified [43] using the forward primer LTR0-Biotin: 5'-agttaccagagtcacacaacagacg-3' (5' Biotin). Amplified DNA was purified using streptavidin-coated magnetic beads which bind the biotinylated LTR0 primer (Dynal magnetic beads; Invitrogen, according to the manufacturer's instructions). A second strand was synthesized using random hexamer primers (Roche), 2 U Klenow fragment (Roche) and 250 μM dNTP. To further amplify the LTR/genomic border, a second (nested) linear amplification was carried out using forward primers in the LTR of the integrated lentivirus (LTR1-Biotin: 5'-taagcagtgggttccctagttagc-3'). DNA was purified and digested as above and ligated to a double stranded MseI adapter (ABI), yielding a double stranded DNA fragment containing the LTR/genomic border with known ends. To further amplify these fragments, two sequential PCRs using forward primers in the LTR of the integrated virus (1^st ^PCR using primer LTR1: 5'-taagcagtgggttccctagttagc-3', 2^nd ^PCR using primer LTR3: 5'-cagagagctcccaggctcag-3'), and a reverse primer in the MseI adapter (5'-ggtcagatgagtcctgagtaa-3') were carried out. PCR products were separated on 3% agarose gels (Biozyme Sieve 3:1), extracted and purified using the Qiaquick Gel Extraction Kit (Qiagen), and then sequenced using primer LTR2: 5'-ccaggctcagatctggtctaac-3'. PCRs, capillary electrophoresis and sequencing were carried out by Services in Molecular Biology GmbH, Berlin.

### Cell culture and bacterial culture

Cell lines were grown in RPMI (AGS and L929) or DMEM (A549) supplemented with 10% fetal calf serum (FCS), 2 mM L-glutamate and 1 mM NaPyruvate (DMEM only). *H. pylori *clinical isolate P1 and deletion mutants thereof were routinely cultured on horse serum agar plates under microairophilic conditions, as described previously [44].

### Automated microscopy and image analysis

Cells were seeded in 96-well plates, one or two days prior to activation to obtain a confluency of approximately 70%. Before activation, medium was replaced by fresh medium (50 μl per well). For activation, another 50 μl of medium containing recombinant human TNFα (BD Pharmingen, 4-12 × 10^8 ^units/mg), recombinant IL-1β (Strathmann Biotech, 1 × 10^7 ^units/mg), LPS from *Salmonella typhosa *(Sigma), or *H. pylori *strain P1 at the appropriate concentration was added. After the respective incubation time, cells were fixed with 100% ice-cold methanol, stained with Hoechst 33342 (2 μg/ml) and stored in phosphate buffered saline (PBS) containing 0.1% NaN_3_. Four images per well were taken by the automated microscopy system Scan^R (Olympus) using autofocus on nuclei. Images were subsequently analyzed by Scan^R software (Olympus) and quantification of p65-translocation was carried out using a modified existing protocol [20]. Here, nuclear areas were identified by Hoechst staining and around every nucleus a one-pixel-wide cytoplasmic area was set. To define compartments as accurately as possible, the nuclear area was eroded by two pixels and the cytoplasmic area was distanced by one pixel from the nucleus (see Figure 2). In the case of L929, the nuclear region was eroded by three pixels and the distance from the cytosol was two pixels. While the setting of subcellular regions was similar to an assay described previously for cells stained with anti-p65 antibodies [20], the analysis of p65-translocation was based on a different strategy. Here, we used software to depict cells in dot plots and set the regions as shown in Figure 2. Subsequently, gates were defined: cells in regions R01, R02 and R03 were defined as cells with mainly nuclear p65-GFP whereas cells in regions R01, R02 and R04 were defined as cells with mainly cytoplasmic p65-GFP. Numbers of cells with nuclear or cytoplasmic p65-GFP in every well were counted by the software. The percentage of cells with nuclear p65-GFP per well were calculated using Microsoft Excel (Percentage of cells with nuclear p65-GFP = cells with nuclear p65-GFP/sum of cells with nuclear and cytoplasmic p65-GFP × 100). Definition of regions and gating was optimized for every cell line and the same assay was used for every experiment. Graphs were compiled using Microsoft Excel or Sigma Plot 8.0.

### Live-cell microscopy

Cells were grown in 3.5 cm^2 ^glass bottom dishes (MatTek). Before infection, medium was changed to medium without phenolred (Invitrogen). Bacteria were harvested in the same medium, stained with Syto 61 (Molecular Probes) according to the manufacturer's recommendations and washed three times. The staining of bacteria fades after some minutes. Images were acquired every two minutes. Microscopy was carried out in a humidified incubator (37°C, 5% CO_2_) using the VT-Infinity system (Visitron Systems). The system is compiled of an Olympus IX81 (Olympus, Japan), VT-Infinity galvo scanner confocal head (Visitron Systems) and a Hamamatsu C9100-02 CCD camera (Hamamatsu Photonics K.K). Images were acquired and processed using MetaMorph (Universal Imaging Corporation) software.

### Immunoblotting

Proteins from lysed cells were solubilized in Laemmli buffer, separated by SDS-PAGE, transferred to PVDF membranes, probed with p65 (Santy Cruz) primary antibodies and HRP-conjugated secondary antibodies (Amersham), and detected with ECL reagent (ICN). Signals were visualized on a phosphorimager (Phosphorimager, FLA-3000 Series; Fuji), and band intensities quantified by 1D evaluation using AIDA software (Raytest). Expression levels were calculated as the mean of five independent experiments.

## Authors' contributions

SB and TFM conceived the project; SB and BB performed experiments; SH and SB developed automated microscopic assay; SB, SH, NM and JS analyzed the data; JS developed mathematical model; SB, LAO, JS and TFM wrote the paper. All authors have read and approved the final manuscript.

## Supplementary Material

Additional file 1 **p65-GFP expression levels in monoclonal cell lines**. Western blot of cell lines expressing p65-GFP and their parental cell lines. Lower panel for A549 SIB01 shows digital enhancement of the upper panel. This blot is representative of at least five independent experiments. Click here for file

Additional file 2 **Lentivirus integration sites**. DNA was extracted from the A549 SIB01 and AGS SIB02 (human) as well as the L929 SIB02 (murine) cell lines and used as a template for PCR amplification (see Methods) of the long-terminal repeat (LTR) region (which borders the viral integration site) of the integrated lentivirus. Six of the resulting PCR products were successfully sequenced. The identified sequences and the integration sites according to BLAST alignments are listed. Click here for file

Additional file 3 **Translocation assays of a) AGS SIB02 and b) L929 SIB01 using Scan^R analysis**. Cells were seeded on 96-well plates, activated with TNFα (10 ng/ml), fixed, stained with Hoechst 33342 and analyzed with automated microscopy. Scatter plots as depicted by the analysis software are shown. Cells are gated for circularity and size (Region R01), intensity of GFP and standard deviation of GFP intensity (Region R02) and the ratio of nuclear to cytoplasmic GFP intensity (Region R03 or R04). Cells in regions R01 and R02 are classified as active or inactive according to nuclear and cytoplasmic GFP intensity (Region R03 or R04). Cells with nuclear p65-GFP are also in region R03, whereas cells with mainly cytoplasmic p65-GFP are also in gate R04. Click here for file

Additional file 4 **Comparison of the threshold-based translocation assay and the distribution of translocation in a cell population**. Reporter cell lines were activated with TNFα (10 ng/ml) for the indicated time, fixed, stained with Hoechst and analyzed with automated microscopy. **Top four rows **show indicated parameters for all cells in one well per time point (approximately 300-1000 cells). **Bottom row **shows the results of the threshold-based translocation assay shown in Figure 2 and Additional file 3. Error bars = SD of duplicates. Click here for file

Additional file 5 **NF-κB activation and kinetics. a) **Comparison of NF-κB activation in parental, non-transduced cell lines with p65-GFP-expressing cell lines. Reporter and parental cell lines respond similarly to a NF-κB stimulus. TNFα (0.5 and 10 ng/ml) was added to reporter and parental cell lines for the indicated times. Degradation of IκBα and actin (control) was analyzed by Western blot. Blots are representative of at least three independent experiments. **b) **Kinetics of p65-GFP nuclear translocation corresponds to IκBα degradation. The reporter cell lines were activated with TNFα (10 ng/ml or 0.5 ng/ml) for the stated time, fixed, stained with Hoechst 33342 and analyzed with automated microscopy and the Scan^R analysis software. For software analysis, individually optimized setups were used for each cell line as shown in Figure 2 and Additional file 3. Error bars = SD of experiment performed in triplicates. Results are representative of three independent experiments. Click here for file

Additional file 6 **NF-κB activation induced by *H. pylori *depends on a functional TFSS but not on CagA or a putative secreted factor in the supernatant**. **a) **p65-GFP-expressing AGS SIB02 cells were infected with the indicated strains at an MOI of 10, fixed after the indicated time and analyzed by automated microscopy. P1 is the wild type strain, P1Δ*cagA *does not express the bacterial effector CagA and P1Δ*virB11 *does not express VirB11, a protein essential for the function of the TFSS. **b) **Supernatants (SN) were collected from cells that were either non-infected or infected with the wild type *H. pylori *at an MOI of 100 for 6 h. After the SN was cleared from remaining bacteria by a passage through a 0.2 μm filter, new AGS SIB02 cells were stimulated either with control medium containing 10 ng/ml TNFα, the SN from the non-infected cells or the SN from the infected cells. Error bars = SD of experiment performed in triplicates. Click here for file

Additional file 7 **Oscillations of p65 nuclear translocation within single cells after infection with *H. pylori***. p65-GFP-expressing AGS SIB02 cells were infected with *H. pylori*, analyzed by confocal live-cell microscopy and nuclear translocation of p65-GFP was quantified by Metamorph software. Each graph shows the average intensity of GFP in a representative nuclear region of a single cell, to which one or more bacteria have attached, at different time points. Colors indicate four separate experiments. Dashed lines at peaks indicate points used for measurement of peak intervals shown in Figure 4d. Click here for file

Additional file 8 **Modeling methods**. Methods for statistical analysis and description of mathematical modeling Click here for file

## References

[B1] Hacker H Karin M Regulation and function of IKK and IKK-related kinases Sci STKE 2006 357 re13 10.1126/stke.3572006re13 17047224

[B2] Hoffmann A Baltimore D Circuitry of nuclear factor κB signaling Immunol Rev 2006 210 171 186 10.1111/j.0105-2896.2006.00375.x 16623771

[B3] Li Q Withoff S Verma IM Inflammation-associated cancer: NF-κB is the lynchpin Trends Immunol 2005 26 318 325 10.1016/j.it.2005.04.003 15922948

[B4] Karin M Greten FR NF-κB: linking inflammation and immunity to cancer development and progression Nat Rev Immunol 2005 5 749 759 10.1038/nri1703 16175180

[B5] Hayden MS Ghosh S Shared principles in NF-κB signaling Cell 2008 132 344 362 10.1016/j.cell.2008.01.020 18267068

[B6] Hoffmann A Natoli G Ghosh G Transcriptional regulation via the NF-κB signaling module Oncogene 2006 25 6706 6716 10.1038/sj.onc.1209933 17072323

[B7] Wertz IE Dixit VM Ubiquitin-mediated regulation of TNFR1 signaling Cytokine Growth Factor Rev 2008 19 313 324 10.1016/j.cytogfr.2008.04.014 18515172

[B8] Nelson DE Ihekwaba AE Elliott M Johnson JR Gibney CA Foreman BE Nelson G See V Horton CA Spiller DG Edwards SW McDowell HP Unitt JF Sullivan E Grimley R Benson N Broomhead D Kell DB White MR Oscillations in NF-κB signaling control the dynamics of gene expression Science 2004 306 704 708 10.1126/science.1099962 15499023

[B9] Ashall L Horton CA Nelson DE Paszek P Harper C Sillitoe K Ryan S Spiller DG Unitt JF Broomhead DS Kell DB Rand DA Sée V White MRH Pulsatile stimulation determines timing and specificity of NF-κB-dependent transcription Science 2009 324 242 10.1126/science.1164860 19359585 PMC2785900

[B10] Sung MH Salvatore L De Lorenzi R Indrawan A Pasparakis M Hager GL Bianchi ME Agresti A Sustained oscillations of NF-kappaB produce distinct genome scanning and gene expression profiles PLoS One 2009 4 e7163 10.1371/journal.pone.0007163 19787057 PMC2747007

[B11] Lee TK Denny EM Sanghvi JC Gaston JE Maynard ND Hughey JJ Covert MW A noisy paracrine signal determines the cellular NF-kappaB response to lipopolysaccharide Sci Signal 2009 2 ra65 10.1126/scisignal.2000599 19843957 PMC2778577

[B12] Hoffmann A Levchenko A Scott ML Baltimore D The IκB- NF-κB signaling module: temporal control and selective gene activation Science 2002 298 1241 1245 10.1126/science.1071914 12424381

[B13] Bosisio D Marazzi I Agresti A Shimizu N Bianchi ME Natoli G A hyper-dynamic equilibrium between promoter-bound and nucleoplasmic dimers controls NF-kappaB-dependent gene activity EMBO J 2006 25 798 810 10.1038/sj.emboj.7600977 16467852 PMC1383558

[B14] Nelson G Paraoan L Spiller DG Wilde GJC Browne MA Djali PK Unitt JF Sullivan E Floettmann E White MRH Multi-parameter analysis of the kinetics of NF-κB signalling and transcription in single living cells J Cell Sci 2002 115 1137 1148 11884514 10.1242/jcs.115.6.1137

[B15] Covert MW Leung TH Gaston JE Baltimore D Achieving stability of lipopolysaccharide-induced NF-κB activation Science 2005 309 1854 1857 10.1126/science.1112304 16166516

[B16] Werner SL Barken D Hoffmann A Stimulus specificity of gene expression programs determined by temporal control of IKK activity Science 2005 309 1857 1861 10.1126/science.1113319 16166517

[B17] Sillitoe K Horton C Spiller DG White MR Single-cell time-lapse imaging of the dynamic control of NF-κB signalling Biochem Soc Trans 2007 35 263 266 10.1042/BST0350263 17371255

[B18] Kearns JD Basak S Werner SL Huang CS Hoffmann A IκBε provides negative feedback to control NF-κB oscillations, signaling dynamics, and inflammatory gene expression J Cell Biol 2006 173 659 664 10.1083/jcb.200510155 16735576 PMC2063883

[B19] Sato TK Yamada RG Ukai H Baggs JE Miraglia LJ Kobayashi TJ Welsh DK Kay SA Ueda HR Hogenesch JB Feedback repression is required for mammalian circadian clock function Nat Genet 2006 38 312 9 10.1038/ng1745 16474406 PMC1994933

[B20] Ding GJF Fischer PA Boltz RC Schmidt JA Colaianne JJ Gough A Rubin RA Miller DK Characterization and quantitation of NF-κB nuclear translocation induced by interleukin-1 and tumor necrosis factor-alpha. Development and use of a high capacity fluorescence cytometric system J Biol Chem 1998 273 28897 28905 10.1074/jbc.273.44.28897 9786892

[B21] Ihekwaba AE Wilkinson SJ Waithe D Broomhead DS Li P Grimley RL Benson N Bridging the gap between in silico and cell-based analysis of the nuclear factor-kappaB signaling pathway by in vitro studies of IKK2 FEBS J 2007 274 1678 90 10.1111/j.1742-4658.2007.05713.x 17313484

[B22] Vakkila J DeMarco RA Lotze MT Imaging analysis of STAT1 and NF-kappaB translocation in dendritic cells at the single cell level J Immunol Methods 2004 294 123 34 10.1016/j.jim.2004.09.007 15604022

[B23] Birbach A Gold P Binder BR Hofer E de Martin R Schmid JA Signaling molecules of the NF-κB pathway shuttle constitutively between cytoplasm and nucleus J Biol Chem 2002 277 10842 10851 10.1074/jbc.M112475200 11801607

[B24] Carlotti F Chapman R Dower SK Qwarnstrom EE Activation of nuclear factor κB in single living cells. Dependence of nuclear translocation and anti-apoptotic function on EGFPRELA concentration J Biol Chem 1999 274 37941 37949 10.1074/jbc.274.53.37941 10608861

[B25] Carlotti F Dower SK Qwarnstrom EE Dynamic shuttling of NF-κB between the nucleus and cytoplasm as a consequence of inhibitor dissociation J Biol Chem 2000 275 41028 41034 10.1074/jbc.M006179200 11024020

[B26] Schmid JA Birbach A Hofer-Warbinek R Pengg M Burner U Furtmüller PG Binder BR de Martin R Dynamics of NF-κB and IκBα studied with green fluorescent protein (GFP) fusion proteins J Biol Chem 2000 275 17035 17042 10.1074/jbc.M000291200 10747893

[B27] Barken D Wang CJ Kearns J Cheong R Hoffmann A Comment on "Oscillations in NF-κB signaling control the dynamics of gene expression" Science 2005 308 52 10.1126/science.1107904 15802586 PMC2821939

[B28] Nelson DE Horton CA See V Johnson JR Nelson G Spiller DG Kell DB White MRH Response to Comment on "Oscillations in NF-κB signaling control the dynamics of gene expression" Science 2005 308 52 10.1126/science.110790415802586

[B29] Viala J Chaput C Boneca IG Cardona A Girardin SE Moran AP Athman R Mémet S Huerre MR Coyle AJ DiStefano PS Sansonetti PJ Labigne A Bertin J Philpott DJ Ferrero RL Nod1 responds to peptidoglycan delivered by the *Helicobacter pylori *cag pathogenicity island Nat Immunol 2004 5 1166 74 10.1038/ni1131 15489856

[B30] Fischer W Püls J Buhrdorf R Gebert B Odenbreit S Haas R Systematic mutagenesis of the *Helicobacter pylori *cag pathogenicity island: essential genes for CagA translocation in host cells and induction of interleukin-8 Mol Microbiol 2001 42 1337 48 10.1046/j.1365-2958.2001.02714.x 11886563

[B31] Maeda S Akanuma M Mitsuno Y Hirata Y Ogura K Yoshida H Shiratori Y Omata M Distinct mechanism of *Helicobacter pylori*-mediated NF-kappa B activation between gastric cancer cells and monocytic cells J Biol Chem 2001 276 44856 64 10.1074/jbc.M105381200 11546774

[B32] Bauer B Moese S Bartfeld S Meyer TF Selbach M Analysis of cell type-specific responses mediated by the type IV secretion system of *Helicobacter pylori* Infect Immun 2005 73 4643 52 10.1128/IAI.73.8.4643-4652.2005 16040977 PMC1201271

[B33] Tsai TY-C Choi YS Ma W Pomerening JR Tang C Ferrell JE Jr Robust, tunable biological oscillations from interlinked positive and negative feedback loops Science 2008 321 126 129 10.1126/science.1156951 18599789 PMC2728800

[B34] Becskei A Boselli MG van Oudenaarden A Amplitude control of cell-cycle waves by nuclear import Nat Cell Biol 2004 6 451 457 10.1038/ncb1124 15107861

[B35] Htun H Barsony J Renyi I Gould DL Hager GL Visualization of glucocorticoid receptor translocation and intranuclear organization in living cells with a green fluorescent protein chimera Proc Natl Acad Sci USA 1996 93 4845 4850 10.1073/pnas.93.10.4845 8643491 PMC39367

[B36] Jacquet M Renault G Lallet S De Mey J Goldbeter A Oscillatory nucleocytoplasmic shuttling of the general stress response transcriptional activators Msn2 and Msn4 in *Saccharomyces cerevisiae* J Cell Biol 2003 161 497 505 10.1083/jcb.200303030 12732613 PMC2172953

[B37] Lahav G Rosenfeld N Sigal A Geva-Zatorsky N Levine AJ Elowitz MB Alon U Dynamics of the p53-Mdm2 feedback loop in individual cells Nat Genet 2004 36 147 150 10.1038/ng1293 14730303

[B38] Shibasaki F Price ER Milan D McKeon F Role of kinases and the phosphatase calcineurin in the nuclear shuttling of transcription factor NF-AT4 Nature 1996 382 370 373 10.1038/382370a0 8684469

[B39] Cheong R Bergmann A Werner SL Regal J Hoffmann A Levchenko A Transient IκB kinase activity mediates temporal NF-κB dynamics in response to a wide range of tumor necrosis factor-alpha doses J Biol Chem 2006 281 2945 2950 10.1074/jbc.M510085200 16321974

[B40] Or-Guil M Wittenbrink N Weiser AA Schuchhardt J Recirculation of germinal center B cells: a multilevel selection strategy for antibody maturation Immunol Rev 2007 216 130 41 17367339 10.1111/j.1600-065X.2007.00507.x

[B41] Karin M Yamamoto Y Wang QM The IKK NF-κB system: a treasure trove for drug development Nat Rev Drug Discov 2004 3 17 26 10.1038/nrd1279 14708018

[B42] Bartfeld S Engels C Bartfeld S Engels C Bauer B Aurass P Flieger A Brüggemann H Meyer TF Temporal resolution of two tracked NF-kappaB activation by *Legionella pneumophila* Cell Microbiol 2009 11 1638 51 10.1111/j.1462-5822.2009.01354.x 19573161

[B43] Schmidt M Zickler P Hoffmann G Haas S Wissler M Muessig A Tisdale JF Kuramoto K Andrews RG Wu T Kiem HP Dunbar CE von Kalle C Polyclonal long-term repopulating stem cell clones in a primate model Blood 2002 100 2737 43 10.1182/blood-2002-02-0407 12351380

[B44] Backert S Ziska E Brinkmann V Zimny-Arndt U Fauconnier A Jungblut PR Naumann M Meyer TF Translocation of the *Helicobacter pylori *CagA protein in gastric epithelial cells by a type IV secretion apparatus Cell Microbiol 2000 2 155 164 10.1046/j.1462-5822.2000.00043.x 11207572

