# Supplementary figures and images for: High-throughput and single-cell imaging of NF-κB oscillations using monoclonal cell lines
Source: BMC Cell Biol. 2010 Mar 16;11:21. doi: 10.1186/1471-2121-11-21 (PMC2848210; doi:10.1186/1471-2121-11-21)

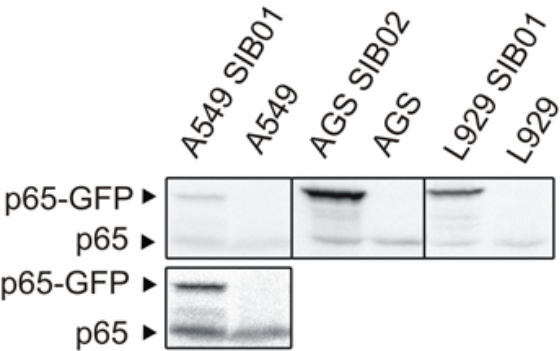

Supplement: Additional file 1 — p65-GFP expression levels in monoclonal cell lines. Western blot of cell lines expressing p65-GFP and their parental cell lines. Lower panel for A549 SIB01 shows digital enhancement of the upper panel. This blot is representative of at least five independent experiments. [file 1471-2121-11-21-S1.PDF]

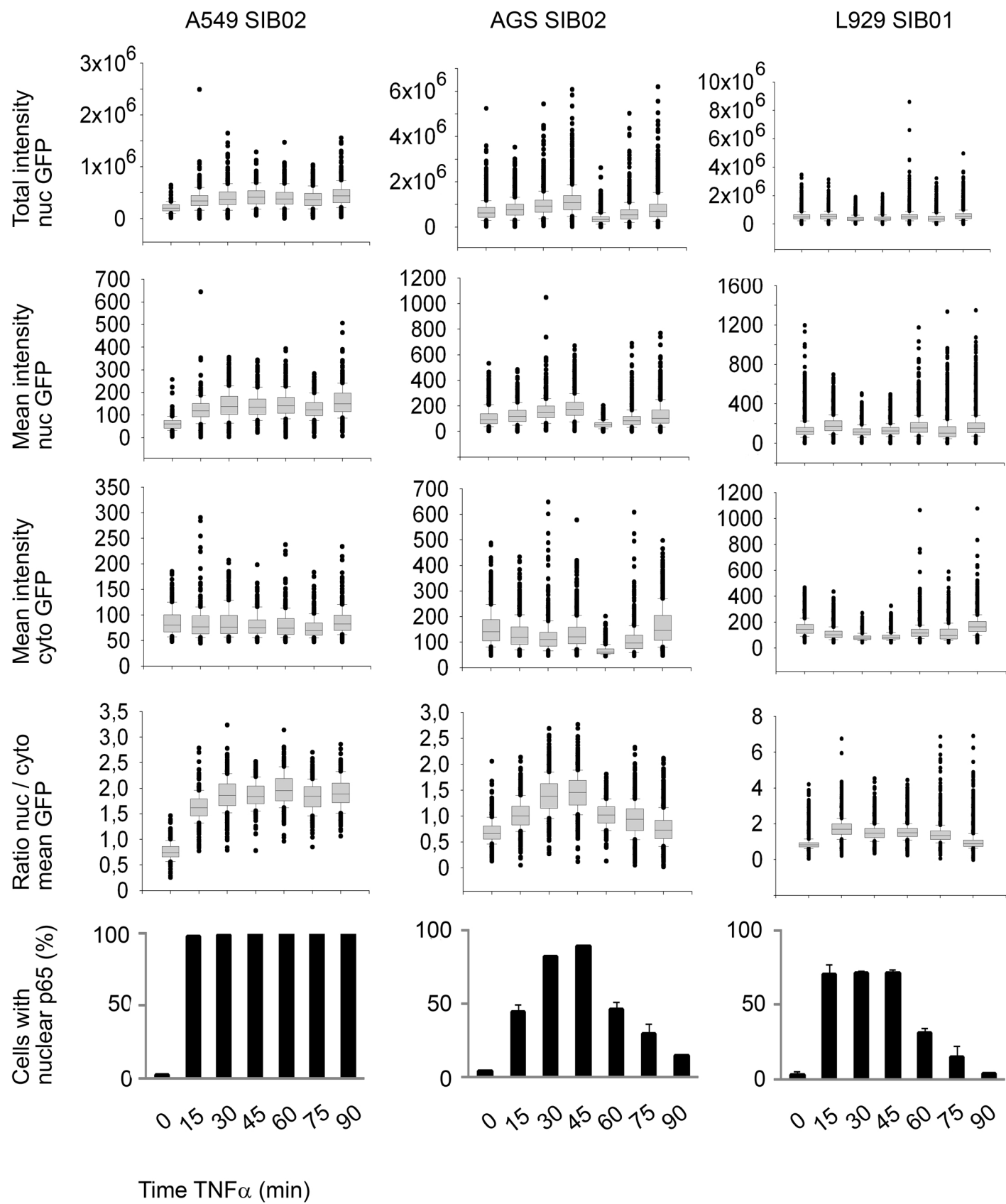

Supplement: Additional file 4 — Comparison of the threshold-based translocation assay and the distribution of translocation in a cell population. Reporter cell lines were activated with TNFα (10 ng/ml) for the indicated time, fixed, stained with Hoechst and analyzed with automated microscopy. Top four rows show indicated parameters for all cells in one well per time point (approximately 300-1000 cells). Bottom row shows the results of the threshold-based translocation assay shown in Figure 2 and Additional file 3. Error bars = SD of duplicates. [file 1471-2121-11-21-S4.PDF]

a

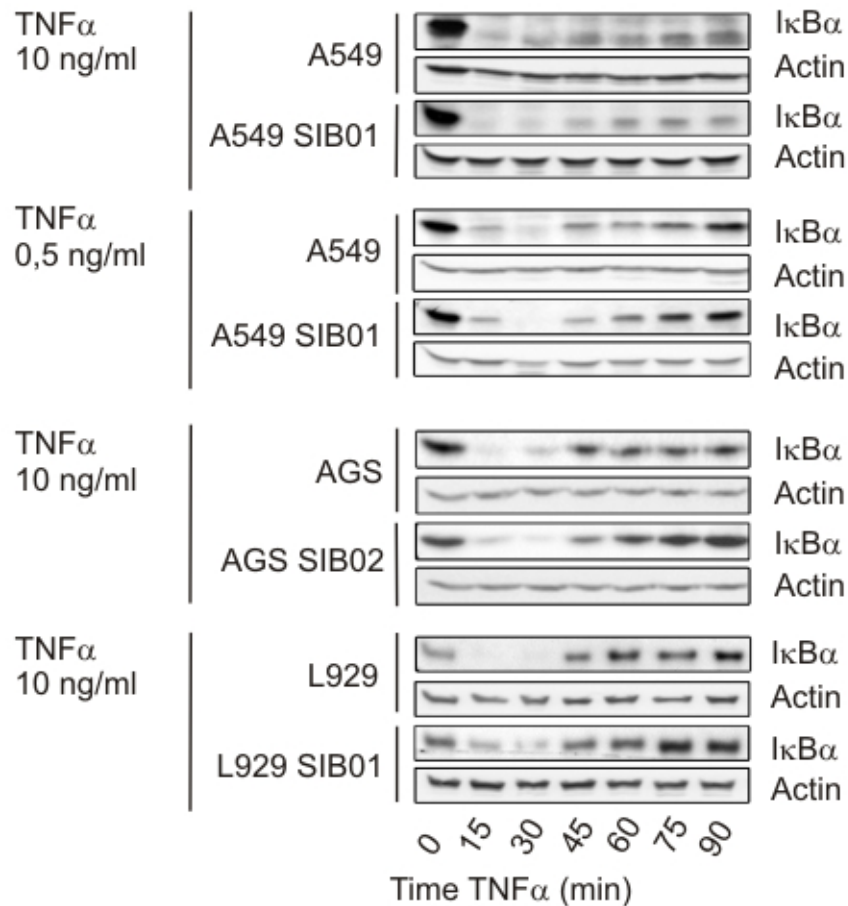

b

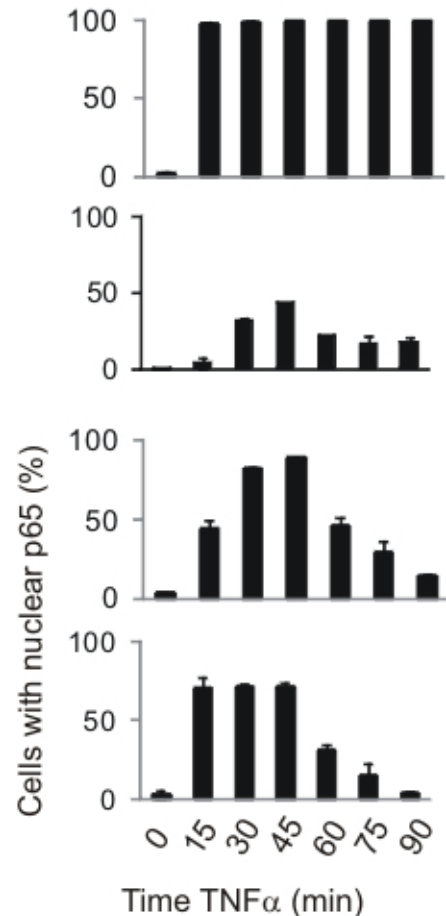

Supplement: Additional file 5 — NF-κB activation and kinetics. a) Comparison of NF-κB activation in parental, non-transduced cell lines with p65-GFP-expressing cell lines. Reporter and parental cell lines respond similarly to a NF-κB stimulus. TNFα (0.5 and 10 ng/ml) was added to reporter and parental cell lines for the indicated times. Degradation of IκBα and actin (control) was analyzed by Western blot. Blots are representative of at least three independent experiments. b) Kinetics of p65-GFP nuclear translocation corresponds to IκBα degradation. The reporter cell lines were activated with TNFα (10 ng/ml or 0.5 ng/ml) for the stated time, fixed, stained with Hoechst 33342 and analyzed with automated microscopy and the Scan^R analysis software. For software analysis, individually optimized setups were used for each cell line as shown in Figure 2 and Additional file 3. Error bars = SD of experiment performed in triplicates. Results are representative of three independent experiments. [file 1471-2121-11-21-S5.PDF]

a

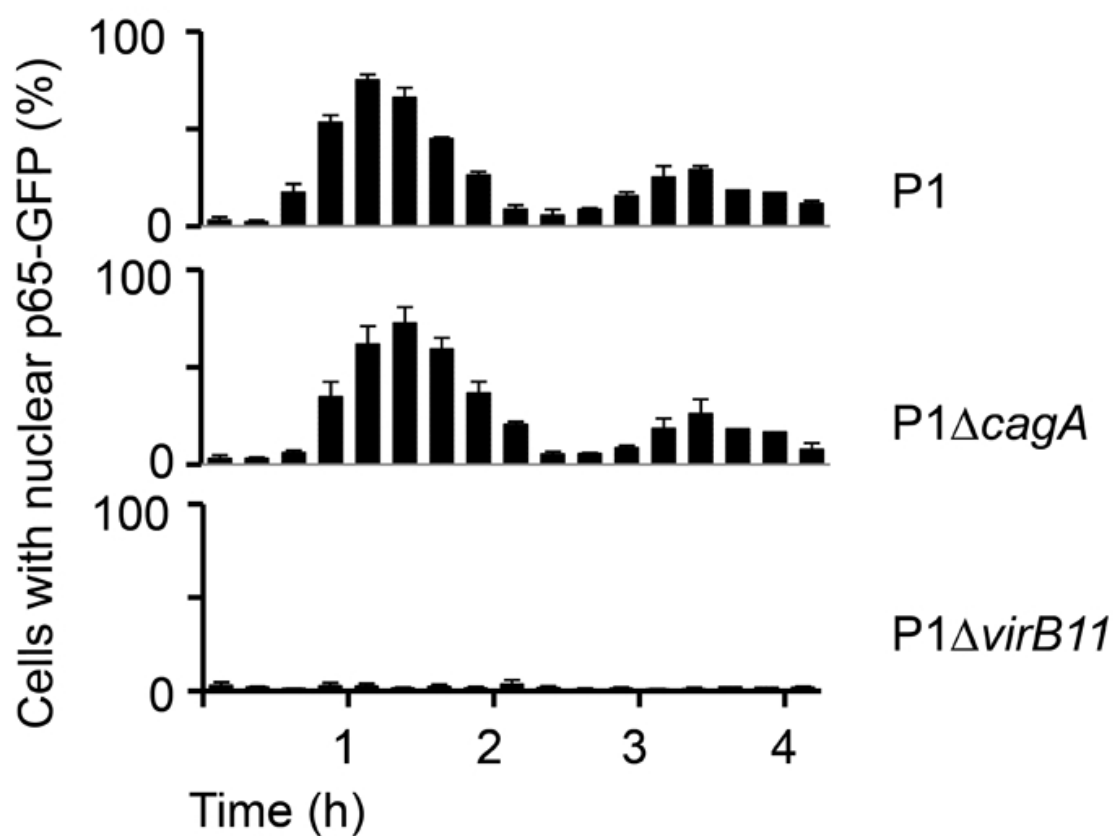

b

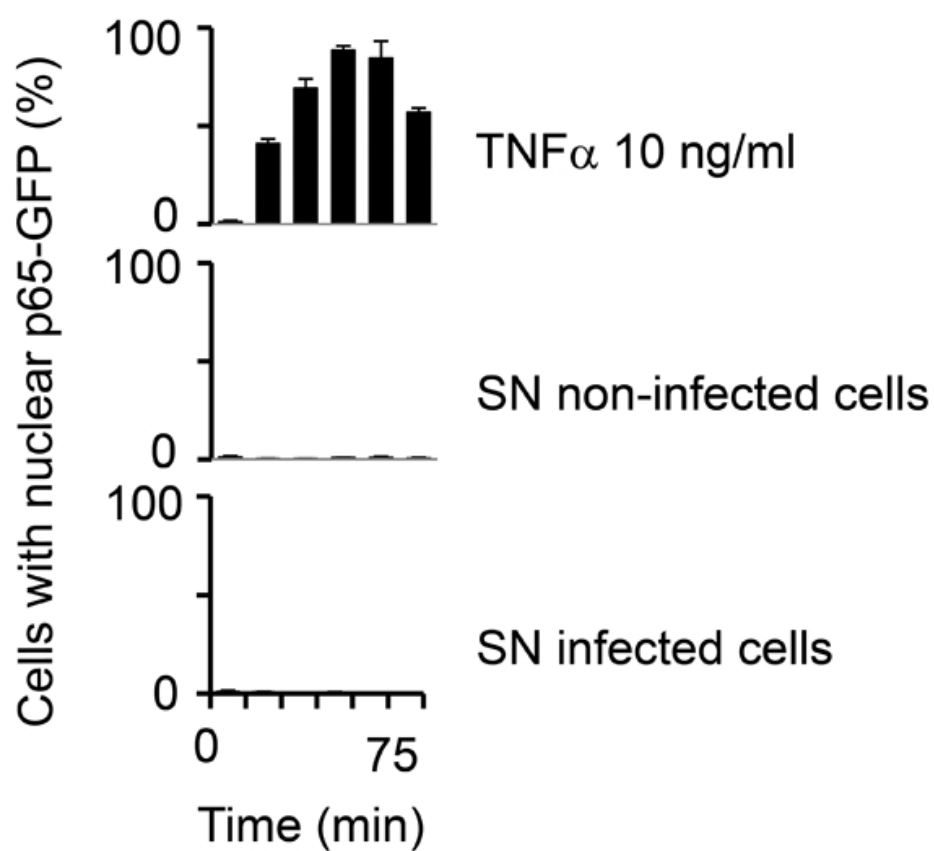

Supplement: Additional file 6 — NF-κB activation induced by H. pylori depends on a functional TFSS but not on CagA or a putative secreted factor in the supernatant. a) p65-GFP-expressing AGS SIB02 cells were infected with the indicated strains at an MOI of 10, fixed after the indicated time and analyzed by automated microscopy. P1 is the wild type strain, P1ΔcagA does not express the bacterial effector CagA and P1ΔvirB11 does not express VirB11, a protein essential for the function of the TFSS. b) Supernatants (SN) were collected from cells that were either non-infected or infected with the wild type H. pylori at an MOI of 100 for 6 h. After the SN was cleared from remaining bacteria by a passage through a 0.2 μm filter, new AGS SIB02 cells were stimulated either with control medium containing 10 ng/ml TNFα, the SN from the non-infected cells or the SN from the infected cells. Error bars = SD of experiment performed in triplicates. [file 1471-2121-11-21-S6.PDF]

Average intensity of GFP in representative nuclear region

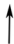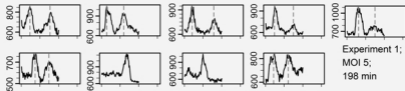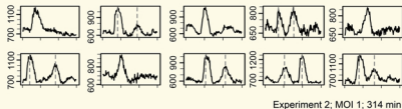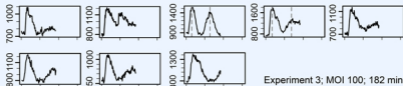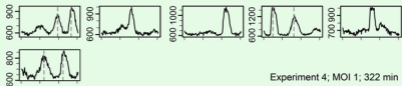

Time (each 0-320 min)

Supplement: Additional file 7 — Oscillations of p65 nuclear translocation within single cells after infection with H. pylori. p65-GFP-expressing AGS SIB02 cells were infected with H. pylori, analyzed by confocal live-cell microscopy and nuclear translocation of p65-GFP was quantified by Metamorph software. Each graph shows the average intensity of GFP in a representative nuclear region of a single cell, to which one or more bacteria have attached, at different time points. Colors indicate four separate experiments. Dashed lines at peaks indicate points used for measurement of peak intervals shown in Figure 4d. [file 1471-2121-11-21-S7.PDF]
