# Supplementary material for: High-throughput and single-cell imaging of NF-κB oscillations using monoclonal cell lines
Source: BMC Cell Biol. 2010 Mar 16;11:21. doi: 10.1186/1471-2121-11-21 (PMC2848210; doi:10.1186/1471-2121-11-21)
Supplement: Additional file 2 — Lentivirus integration sites. DNA was extracted from the A549 SIB01 and AGS SIB02 (human) as well as the L929 SIB02 (murine) cell lines and used as a template for PCR amplification (see Methods) of the long-terminal repeat (LTR) region (which borders the viral integration site) of the integrated lentivirus. Six of the resulting PCR products were successfully sequenced. The identified sequences and the integration sites according to BLAST alignments are listed. [file 1471-2121-11-21-S2.PDF]

Cell line	Sequence of PCR product	Integration site according to BLAST alignment
A549 SIB01	CTCTACCAAAATGCTTGTTATATGATGTAACTCTCAAAATGCTACCTGATTCTAAAGAATGCATTTGCTCATAAAACAATGAGAGGTGGCAGGTGCATTGTTCATTCTCTTGGATTTCCAACAAACATTTAATATGCTTTTCTGAATTGTTACTTTCCTTGTCTATCTTGT	Homo sapiens chromosome 12. NT_009755.19. Position 7510752 to 7510922. Features in this sequence: transmembrane protein 132D; TMEM132D. Intron 2.
AGS SIB02	TTGATGAGTCCTGAGTAAAGGAGAAAAGAGCCCACTGCAGATTTTCAAGCCAATTCCATCCCTTCAAGCCCACCCCCACCTTTGCACACACGCAAACACACACGTGCACACACACACACACATCACTGCTGCCACCACCACCACTGCCACCACCACCATTTTCTCTCCTCTCATATAAATAACTGACAATGTTCCCCTGAATATAAACCACATGGACCCACTAAAGCTACATGCAAATTGACAGAAGATTCCAATCCTGCATCATGCCAGAGAGCTCAAGGAAGAAAGTCCTATCCTGCGGTCTCAGTTGGCAGGAGCCTCTGTCCTACAATTTCCTGACCCGGTCAAAGCAACCTTATAACCTCACGGGCAAAGACATGAGATGAATCAAGAGGGTTACTCAAGACTCATC	Homo sapiens chromosome 2. NT_022184.15. Position 39155292 to 39155675. No features in this sequence. Features flanking this sequence: 856359 bp at 5' side: similar to hCG1815165. 345912 bp at 3' side: B-cell CLL/lymphoma 11A isoform 3.
AGS SIB02	ACCTATCTGAAAAAAAtAACAATAATTaaaTAAATAAATAAAGCCTTGGGAAAGCCCGTTGGTAAAAGACACATGTCTGATTTTATCTTTTACGAGCCCCCTTACCCTGTGAGTTTATCAGCCTTACACGGCTAATGCACGGGGGGGGAATAGTTTCTTATTATGGTGTCCTTTTTCCCCTAAGACTGAAAAAACC	Homo sapiens chromosome 2. NT_022184.15. Position 21913858-21914014. No features at this site. Features flanking this sequence: 72295 bp at 5' side: 3-hydroxyanthranilate 3,4-dioxygenase. 359331 bp at 3' side: zinc finger protein 36, C3H type-like 2
L929 SIB01	ACATCACGTTGGGTAACATTGCTTTTCAAAAACATTTTGAGACTATACGTAAATGATCtTATTgATTTTGTATATATGAGTACACCACCGTTGCTGaTCTTCAGACAGACTA	Mus musculus chromosome 17. NT_039649.7. Position 52357582-52357685. No features in this sequence. Features flanking this part of sequence: 41915 bp at 5' side: Rab31-like. 9702 bp at 3' side: protein phosphatase 4, regulatory subunit 1.
L929 SIB01	TGTGCGGTGGGGTGGGAAGTCCCTCTACTCGTATCAGACACCTGGGAGATGATACAGTTTCTGGACTGGACAAGAGTGACTTTAAGTGAAATGCTCATTTCTGGGGAGAGGTTGTTCCTGTTGTTCTTTTACACGAGGTCAATTCTGGGTTAAAAATTTTGACTGTGGGTTAATAACACCATCCCTCCTCTTAACGCCCTGTCTACCTCCTGGAAGTTAACACTATGAGTTACCTCTCCCCACTGTTGAGCATTTCACCTAAAGTCACTCCTGCTCAGTCCTGAAACTCTCTCATCTCCCAGGTCTCTGCTACTTTCTAGAGGGACTTCCCACCCACCCCACACTTTCTGAGCCTGGGAGCTCTCTG	Mus musculus chromosome 19. Position 43272966-43273076. No features in this sequence. Features flanking this part of sequence: 55846 bp at 5' side: similar to rat ribosomal protein L13a. 64525 bp at 3' side: SORCS receptor 1.
L929 SIB01	GGCcAGGGGGCCTCTCCCCTCACCaCCTCATTTGACCTCCCCAGTCCTTGGAATCTAAcTTcTTCTCATTGGCTTCCtCTAGGATAC	Mus musculus chromosome 19. NT_039687.7. Position 38005522- 38005436. Feature in this sequence: Paired box gene 2. Intron 8. Features flanking this part of subject sequence: 119617 bp at 5' side: olfactory receptor 1505. 363523 bp at 3' side: hypothetical protein.
