# Supplementary material for: High-throughput and single-cell imaging of NF-κB oscillations using monoclonal cell lines
Source: BMC Cell Biol. 2010 Mar 16;11:21. doi: 10.1186/1471-2121-11-21 (PMC2848210; doi:10.1186/1471-2121-11-21)
Supplement: Additional file 3 — Translocation assays of a) AGS SIB02 and b) L929 SIB01 using Scan^R analysis. Cells were seeded on 96-well plates, activated with TNFα (10 ng/ml), fixed, stained with Hoechst 33342 and analyzed with automated microscopy. Scatter plots as depicted by the analysis software are shown. Cells are gated for circularity and size (Region R01), intensity of GFP and standard deviation of GFP intensity (Region R02) and the ratio of nuclear to cytoplasmic GFP intensity (Region R03 or R04). Cells in regions R01 and R02 are classified as active or inactive according to nuclear and cytoplasmic GFP intensity (Region R03 or R04). Cells with nuclear p65-GFP are also in region R03, whereas cells with mainly cytoplasmic p65-GFP are also in gate R04. [file 1471-2121-11-21-S3.PDF]

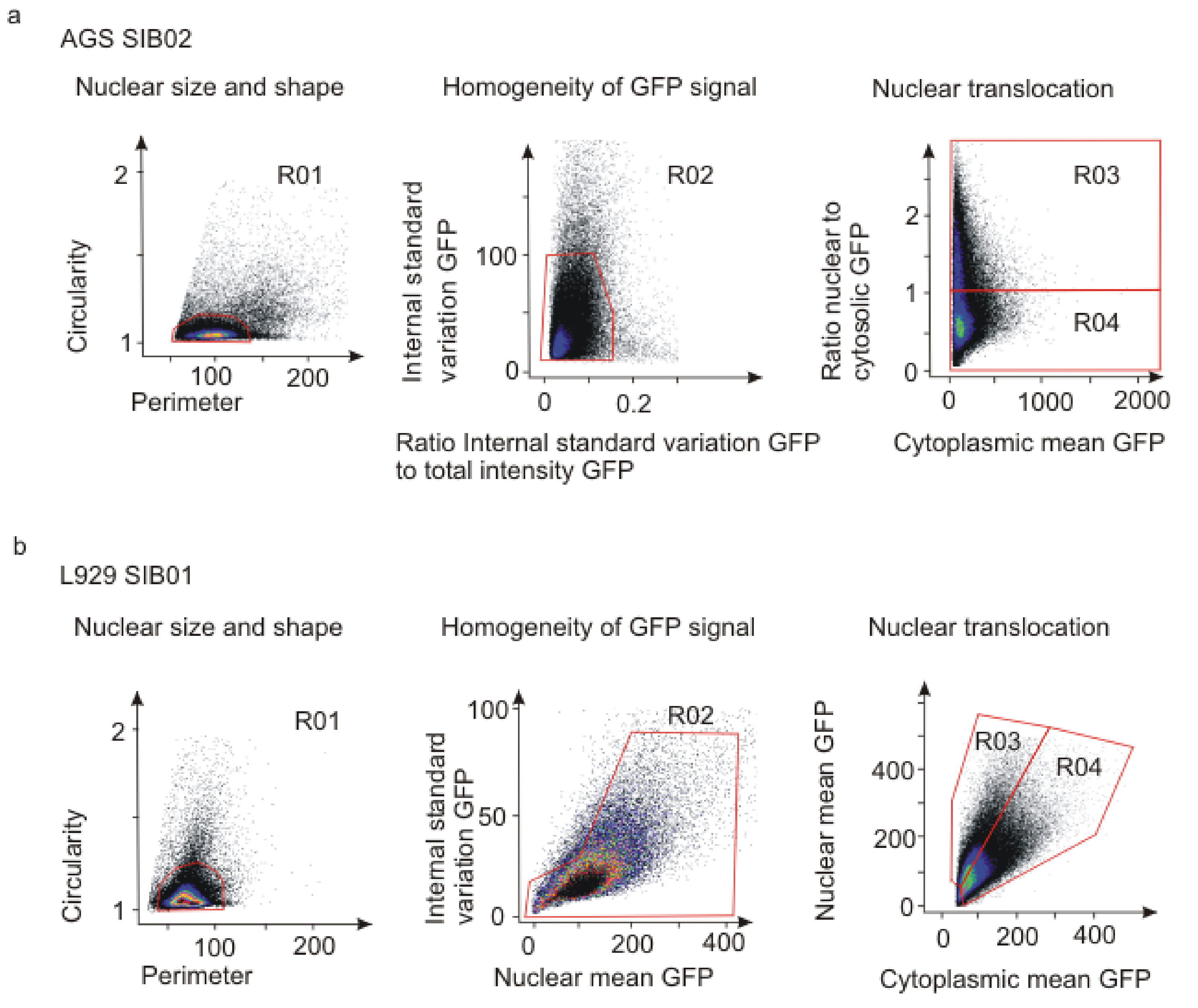
a
AGS SIB02
Nuclear size and shape
Circularity
Perimeter
R01
Homogeneity of GFP signal
Internal standard variation GFP
Ratio Internal standard variation GFP to total intensity GFP
R02
Nuclear translocation
Ratio nuclear to cytosolic GFP
Cytoplasmic mean GFP
R03
R04
b
L929 SIB01
Nuclear size and shape
Circularity
Perimeter
R01
Homogeneity of GFP signal
Internal standard variation GFP
Nuclear mean GFP
R02
Nuclear translocation
Nuclear mean GFP
Cytoplasmic mean GFP
R03
R04
