# Supplementary material for: High-throughput and single-cell imaging of NF-κB oscillations using monoclonal cell lines
Source: BMC Cell Biol. 2010 Mar 16;11:21. doi: 10.1186/1471-2121-11-21 (PMC2848210; doi:10.1186/1471-2121-11-21)
Supplement: Additional file 8 — Modeling methods. Methods for statistical analysis and description of mathematical modeling [file 1471-2121-11-21-S8.PDF]

Methods for statistical analysis

Peak alignment of time series

Data was sampled from different cells at 2 min intervals and stored in a data matrix. Peak alignment was performed for nine cells observed in film 1 of our cell tracking experiments. All time series signals are normalized to the unit interval as defined by their respective minimum and maximum value. A three step algorithm was designed to perform automatic alignment. Step 1: smoothing by applying a moving median of 9. Step 2: detection of ascending flanks by smooth step filter (tanh(-6:6/3)). Step 3: identification of highest peak corresponding to the strongest ascending flank in time series. For final alignment position of ascending flank was shifted to zero.

Determination of peak positions

Starting from the data set comprising 33 time series, a subset of 18 curves with strong multi-modal shape was selected to determine the peak intervals. Pairs of successive maxima were identified by median smoothing and peak finding: a median length of 9 was employed on the data, a set of maximal peaks is found by first iteratively identifying the current maximal peak, then applying an exclusion region of 30 steps around the peak. Only the two highest maxima were used for peak interval determination.

Peak interval histogram and normal approximation

Peak intervals were defined from pairs of successive maxima as identified by the method described above. A corresponding histogram of peak intervals was constructed. Statistics of peak intervals can be summarized by the parameters: mean= 93.78 [min], standard deviation 22.99 [min]. These values were employed for parameterization of a Normal (Gauss) shape curve.

Description of mathematical modeling

Mathematical models have been used as tools to understand NF-κB oscillation[1-4]. While some models try to implement the full complexity of the biological system[1, 4], others follow a reductionist approach focusing on few key mechanisms[2, 3, 5, 6]. We combine here the general principles of canonic NF-κB regulation[7] with concepts for a simplifying representation of transcription regulatory feedback loops using a delay model[5]. As a result, we present here a highly focused three variable kinetic model of NF-κB dynamics, able to reproduce the essential kinetics of our experiment.

The resulting set of differential delay equations has the form

d/dt PD = k1a P*d - k1d PD

d/dt PI = k2a P*I - k2d PI

d/dt I = rho PD(t-tau) - (delta+stimulus(t))*I

P represents nuclear transcription factor p65, I represents the inhibitor IκB, and D represents DNA and the transcription factor binding site regulating IκB transcription. Total p65 and DNA are assumed conserved in our model introducing two constants Ptotal and Dtotal. PI is the inactivated complex of transcription factor and inhibitor, PD is the active complex inducing IκB transcription. Competitive binding is modeled by first order kinetics with constants k1a/d and k2a/d, the delayed action of transcription and translation is modeled as a fixed delay time tau and a net IκB protein production

rate rho. External control is mediated by a degradation rate delta which is affected by a time dependent stimulus. In summary, oscillatory kinetics is resulting from delayed inhibitory auto-regulation of IκB expression. A more detailed discussion of the relation of different oscillation models is given by Tiana and colleagues [6]. Simulations were performed using XPP software (http://www.math.pitt.edu/~bard) and the R statistics software (http://www.r-project.org/). Constants: k1a = 0.0125; k1d = 12.5; k2a = 1250; k2d = 0.125; rho = 0.03125; delta = 0.000125; tau = 40; Ptotal = 100; Dtotal = 1. Function: If (t>0) return 0.3125, else return 0.

Variability on the cell population level was introduced in terms of parameter variations affecting rho (+/- 15%) and tau (+/- 20%) responsible transcription and translation. This variation is summarizing the influence of systemic control on the process of transcription and translation, processes known to be strongly dependent on the internal state of the cellular system. The size of the system was fixed to 500 cells.

Dynamics of the infection process was modeled by making appropriate assumptions on the time dependent occurrence of infection events. Two situations were representatively modeled employing Gamma distributions, which is a reasonable choice for simulating waiting times. The rapid onset situation (MOI=100) is modeled with parameters shape=2 and scale=10, corresponding to a short and intensive volley of infection events. The delayed onset is modeled using parameters shape=8 and scale=10, resulting in a much broader distribution. A more detailed discussion of possible approaches to modeling the infection process will be given elsewhere (Schuchhardt et al. in preparation).

Reference List

1. Hoffmann,A., Levchenko,A., Scott,M.L., & Baltimore,D. The IkappaB-NF-kappaB signaling module: temporal control and selective gene activation. *Science* **298**, 1241-1245 (2002).

2. Krishna,S., Jensen,M.H., & Sneppen,K. Minimal model of spiky oscillations in NF-kappaB signaling. *Proc. Natl. Acad. Sci. U. S. A* **103**, 10840-10845 (2006).

3. Lipniacki,T., Paszek,P., Brasier,A.R., Luxon,B., & Kimmel,M. Mathematical model of NF-kappaB regulatory module. *J. Theor. Biol.* **228**, 195-215 (2004).

4. Werner,S.L., Barken,D., & Hoffmann,A. Stimulus specificity of gene expression programs determined by temporal control of IKK activity. *Science* **309**, 1857-1861 (2005).

5. Monk,N.A. Oscillatory expression of Hes1, p53, and NF-kappaB driven by transcriptional time delays. *Curr. Biol.* **13**, 1409-1413 (2003).

6. Tiana,G., Krishna,S., Pigolotti,S., Jensen,M.H., & Sneppen,K. Oscillations and temporal signalling in cells. *Phys. Biol.* **4**, R1-17 (2007).

7. Hoffmann,A. & Baltimore,D. Circuitry of nuclear factor kappaB signaling. *Immunol. Rev.* **210**, 171-186 (2006).
